# Comparative Binding Analysis of Dipeptidyl Peptidase IV (DPP-4) with Antidiabetic Drugs – An *Ab Initio* Fragment Molecular Orbital Study

**DOI:** 10.1371/journal.pone.0166275

**Published:** 2016-11-10

**Authors:** Sundaram Arulmozhiraja, Naoya Matsuo, Erika Ishitsubo, Seiji Okazaki, Hitoshi Shimano, Hiroaki Tokiwa

**Affiliations:** 1 Department of Chemistry, Rikkyo University, 3-34-1 Nishi-Ikebukuro, Toshima-ku, Tokyo 171–8501, Japan; 2 Research Center for Smart molecules, Rikkyo University, 3-34-1 Nishi-Ikebukuro, Toshima-ku, Tokyo 171–8501, Japan; 3 Department of Internal Medicine (Endocrinology and Metabolism), University of Tsukuba, Tsukuba 305–8575, Japan; 4 AMED-CREST, Tokyo, Japan; Katholieke Universiteit Leuven Rega Institute for Medical Research, BELGIUM

## Abstract

Dipeptidyl peptidase IV (DPP-4) enzyme is responsible for the degradation of incretins that stimulates insulin secretion and hence inhibition of DPP-4 becomes an established approach for the treatment of type 2 diabetics. We studied the interaction between DPP-4 and its inhibitor drugs (sitagliptin **1**, linagliptin **2**, alogliptin **3**, and teneligliptin **4**) quantitatively by using fragment molecular orbital calculations at the RI-MP2/cc-pVDZ level to analyze the inhibitory activities of the drugs. Apart from having common interactions with key residues, inhibitors encompassing the DPP-4 active site extensively interact widely with the hydrophobic pocket by their hydrophobic inhibitor moieties. The cumulative hydrophobic interaction becomes stronger for these inhibitors and hence linagliptin and teneligliptin have larger interaction energies, and consequently higher inhibitory activities, than their alogliptin and sitagliptin counterparts. Though effective interaction for both **2** and **3** is at $S_{2}^{'}$ subsite, **2** has a stronger binding to this subsite interacting with Trp629 and Tyr547 than **3** does. The presence of triazolopiperazine and piperazine moiety in **1** and **4**, respectively, provides the interaction to the S_2_ extensive subsite; however, the latter’s superior inhibitory activity is not only due to a relatively tighter binding to the S_2_ extensive subsite, but also due to the interactions to the S_1_ subsite. The calculated hydrophobic interfragment interaction energies correlate well with the experimental binding affinities (K_D_) and inhibitory activities (IC_50_) of the DPP-4 inhibitors.

## Introduction

Dipeptidyl peptidase IV (DPP-4) is the enzyme responsible for the degradation of incretins such as glucagon-like peptide 1 (GLP-1) and glucose-dependent insulinotropic polypeptide, which stimulate insulin secretion from pancreatic beta cells of the islets of Langerhans and suppress glucagon secretion [1–3]. Since type 2 diabetes is due to insufficient insulin production, inhibition of DPP-4, rather than insulin and its secretalogues, now becomes a well-tolerated therapeutic route for the treatment of type 2 diabetes because it improves plasma glucose metabolism efficiently and safely without hypoglycemia. Subsequently, a new class of oral hypoglycemic agents for the treatment of type 2 diabetes were emerged and these are called DPP-4 inhibitors, which restrain the action of DPP-4 thereby prolonging incretin effect *in vivo* [4].

**Fig 1:**
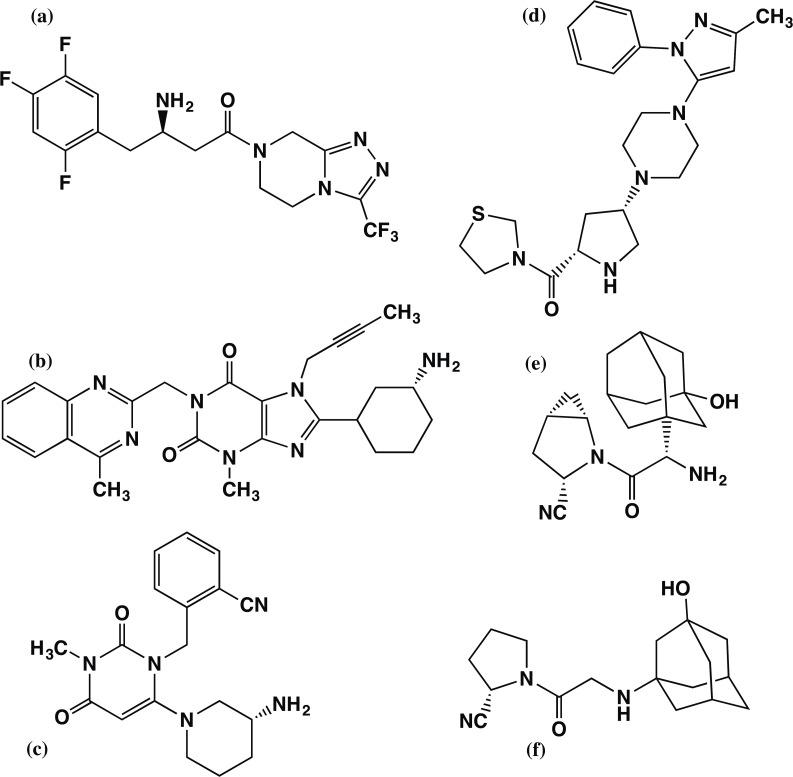
Schematic structure of the DPP-4 inhibitors: (a) sitagliptin, (b) linagliptin, (c) alogliptin, (d) teneligliptin, (e) saxagliptin, and (f) vildagliptin.

X-Ray crystal structures of DPP-4 complex with several inhibitors (sitagliptin **1** [5], linagliptin **2** [6], alogliptin **3** [7], teneligliptin **4**, [8], saxagliptin **5** [9], and vildagliptin **6** [10]) have been reported in recent years. The schematic structures of all these DPP-4 inhibitors are given in Fig 1. Nabeno et al. [10] determined the co-crystal structure of vildagliptin **6** with DPP-4 by X-ray studies and compared the binding modes of all these six launched inhibitors in DPP-4 [10]. These authors also studied the relationship between binding interactions of these six inhibitors with DPP-4 and their inhibitory activities based on structural biology. The interaction between DPP-4 and its inhibitors were qualitatively studied also by Scapin and others [11]. Recently, Schnapp et al. [12] investigated the binding kinetics and thermodynamics of DPP-4 inhibitors using surface plasmon resonance (SPR) and isothermal titration calorimetry (ITC). Nevertheless, there is no report detailing the interactions between the DPP-4 and its inhibitors, especially at molecular level, quantitatively.

The DPP-4 inhibitors bind to the DPP-4-GLP-1 interacting site and hence these inhibitors may be considered as protein-protein interaction (PPI) inhibitors. It has been reported that the evolved PPI inhibitors tend to be larger, highly hydrophobic, very rigid, and contain multiple aromatic rings [13]. Understanding the interaction involving such inhibitors therefore necessitates accurate estimation of hydrophobic interaction energy between the inhibitors and their target proteins. Hence in the present investigation we quantitatively evaluate the interaction energies between DPP-4 and its inhibitors by using the first-principles calculations-based fragment molecular orbital (FMO) quantum-mechanical (QM) method.

The FMO-QM method developed by Kitaura and co-workers [14–16] is a fragmentation method, through which a system is divided into fragments, for example, each amino acid in proteins is considered as a fragment, and the total properties of large molecular systems are derived in a many-body expansion by combining the properties of fragments. In the two-body method, fragments and their pairs (called as ‘dimers’) are calculated. The calculated interfragment pair interaction energies, or simply interfragment interaction energies (IFIEs), are used to analyze the molecular recognition of ligands by proteins. By summing up these IFIEs calculated for individual fragments, overall binding energy between the ligand and protein can be estimated, which can be related to the resultant biological potency of the compound as measured in a biological assay. The correlated FMO calculation provides a complete list of interactions formed between the ligand and protein and so it is suitable for evaluating not only the electrostatic interactions (important in hydrogen-bonding) but also the van der Waals dispersion interactions (hydrophobic interactions) reliably and hence it has been successfully applied to study a lot of protein-ligand interactions by consistently evaluating their binding energies [17–21]. Recently, FMO calculations were made to study protein-peptide interactions to explain the experimental results [22]. In the present work, we use FMO-QM method to quantitatively study the interaction between these DPP-4 inhibitor drugs that are available on the market and the DPP-4. Particular importance is given to study the interactions at a residue level and the results are then used to discuss and analyze the inhibitor activities of the drugs through their structural aspects.

## Methods

We performed *ab initio* FMO calculations to evaluate the interaction energies between various inhibitor drugs with the DPP-4. These non-covalent type endogenous substrates, sitagliptin **1**, linagliptin **2**, alogliptin **3**, and teneligliptin **4**, are the currently worldwide marketed DPP-4 inhibitor drugs for the treatment of type-2 diabetics. The remaining two covalent type drugs (saxagliptin **5** and vildagliptin **6**) were not considered in this FMO study because binding of these drugs requires to overcome the energy barrier for the formation of covalent bond between the nitrile group of their cyanopyrrolidine moieties and the hydroxyl of Ser630 of DPP-4. Evaluating the energy barrier by using the FMO calculation is not a straight forward one.

The crystal structures of the complexes containing DPP-4 and the inhibitors [sitagliptin **1** (PDB ID: 1X70), linagliptin **2** (2RGU), alogliptin **3** (3G0B), teneligliptin **4** (3VJK)] were taken from the Protein Data Bank.

At first, we re-refined and rebuilt the atomic coordinates of the crystal structures of these four DPP-4-inhibitor complexes. The protocols of our re-refinement and re-building the crystal structures of the protein-ligand complex are the following: (i) optimizing the refinement of the target complex structures using *PDB_REDO* web server [23]. This server optimizes various refinement parameters (including *B*-factor weight, X-ray weight, TLS groups, bulk solvent modeling), chooses between an anisotropic or isotropic *B*-factor model, rebuilds side chains in rotamer conformations, flips side chains to optimize hydrogen bond networks, checks peptides for ‘flipping’, re-evaluates the water model, and validates all the present ligands, (ii) deleting the sugar chain coordinates, and (iii) making cycles of *REFMAC* v5.8 [24] maximum-likelihood positional and *B*-factor refinements combined with manual remodeling using *Coot* [25] until the *R* and *R*_free_ factors converged. Default procedures were used for the re-refinement using *REFMAC* program, a part of the *CCP*4 suite [26], in which generation of restraints is an entirely automatic process conducted within *REFMAC* refinement runs when the monomer (ligand) listed in a coordinate PDB file is present in the dictionary with a complete description. Needless to say, complete description of the amino acids is available in this dictionary. The template-restraint library of *CCP*4 contains the necessary cifs (crystallographic information files) of the ligands; however, the cif file for **4** is incomplete and so we used *JLigand* [27], a *CCP4* graphical interface to *REFMAC*, to generate the necessary cif for this gliptin to be used by *REFMAC* for refinement.

Secondly, the missing hydrogen atoms were added by using the Protonate3D module [28] within Molecular Operating Environment (MOE) program [29]. The orientations of the added hydrogen atoms were then optimized by using energy minimization scheme through molecular mechanics calculations utilizing Amber12: EHT force field, which uses Amber12 parameters for macromolecules and extended Huckel theory parameterization for small molecules that takes electronic effects into account, incorporated in MOE program [29]. The final complex structures were then used for the subsequent FMO calculations. It should be mentioned that since all the FMO calculations in the present study were made in vacuum condition, amino groups of the DPP-4 inhibitors (**1**–**3**) were treated as uncharged NH_2_ groups, to avoid overestimation of charge-charge electrostatic interactions.

Finally, all-important FMO calculations were made to study the interactions in all the four selected DPP-4-inhibitor (**1**, **2**, **3**, and **4**) complexes using the PAICS program [30] at the correlated Resolution-of-Identity second-order Moller Plesset (RI-MP2) level [31–33] with the cc-pVDZ basis set [34]. In general, in the FMO calculations, each protein-ligand complex structure is divided into one-residue fragments, with cut-off points at Cα of each residue. Ligand is also considered as a fragment. For the interaction energy calculation, each amino acid residue in the protein is allowed to interact with the ligand. In the first step, each monomer (fragment) is calculated in the Coulomb field exerted by all remaining monomers. This changes the monomer electron densities and hence the coulomb field they determine. Thus, the monomer calculations are repeated self consistently. In the second step, each dimer (pair of fragments) is computed in the Coulomb field exerted by all remaining monomers. By subtracting the two monomer energies from a dimer energy, one obtains the pair interaction or interfragment interaction energy (IFIE). Finally, the total energy of the whole system is constructed from the monomer and the interaction energies. Detailed descriptions of the FMO strategy and methodology can be found in the literature [14–16, 35].

**Fig 2:**
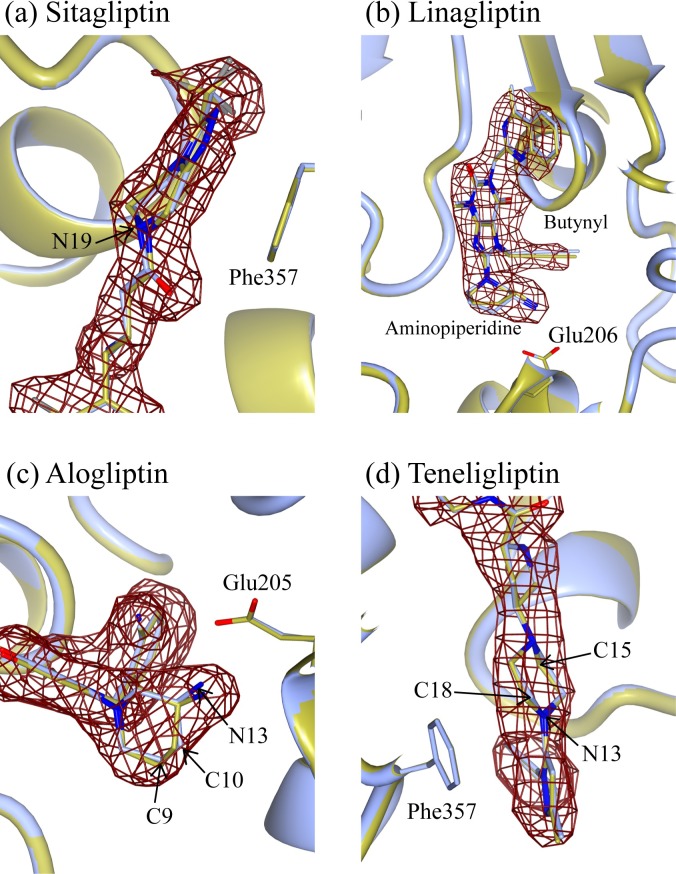
Superposition of the active site of DPP-4-ligand complex structures deposited in Protein databank (ice blue) and the re-refined and re-built (gold). A σ_A_-weighted *F*_O_-*F*_C_ omit map (3.0σ, in brown mesh) is superposed on the inhibitors. (a) Sitagliptin **1**. (b) Linagliptin **2**. (c) Alogliptin **3**. (d) Teneligliptin **4**.

**Fig 3:**
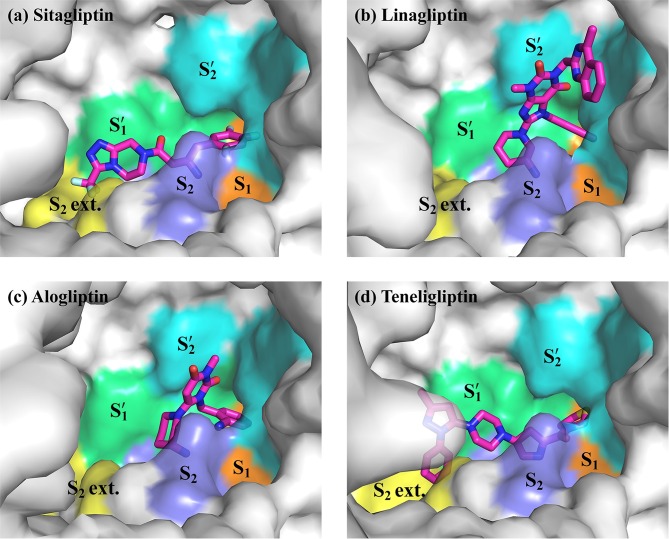
Binding modes of each inhibitor [(a) sitagliptin **1**; (b) linagliptin **2**; (c) alogliptin **3**; (d) teneligliptin **4**] in the active site of DPP-4 complex structure. S_2_ ext. (S_2_ extensive) subsite constructed by Val207, Ser209, Phe357, Arg358, in yellow. S_2_ subsite constructed by Arg125, Phe357, Arg358, Glu205, Glu206, Arg669, in light purple. S_1_ subsite constructed by Ser630, Val656, Trp659, Tyr662, Tyr666, Val711, Asn710, in orange. $S_{1}^{'}$ subsite constructed by Phe357, Tyr547, Pro550, Ser630, Tyr631, Tyr666, in light green. $S_{2}^{'}$ subsite constructed by Tyr547, Trp629, Ser630, His740, in cyan.

To understand the interacting site, the interfacial buried surface area between DPP-4 and the inhibitors were calculated using *AREAIMOL* implemented in the *CCP*4 program suite with a probe radius of 1.4 Å [26]. Fig 2 and Fig 3 were produced using *CCP*4*mg* [36] and PyMOL [37], respectively.

## Results and Discussion

### Improving structures by re-refinement and re-building

Recently, post-deposition optimization is reported to generally improve the protein structures [38] and therefore to improve the qualities of the selected DPP-4 complex structures for the FMO calculations, we re-refined and re-built all these complex structures by using *PDB_REDO* server, *REFMAC* v5.8, and Coot. Three out of four re-refined structures have improved *R*_free_ values (complex structure with linagliptin 0.276 → 0.264, alogliptin 0.242 → 0.260, teneligliptin 0.279 → 0.274, sitagliptin 0.228 → 0.213), which is used for cross-validation [39], despite deleting the coordinates of the sugar chains to simplify the subsequent FMO calculations. Especially, coordinate positions of the inhibitors are improved considerably. The most prominent example is the structure with the inhibitor **1**: the nitrogen atom (N19), of **1**, of the deposited structure (1X70.pdb) has a *sp*^3^ hybridized form, but it becomes a *sp*^2^ hybridized one after the re-refinement, and the omit map of **1** supports the latter form of the nitrogen (see Fig 2A). A root mean square deviation (rmsd) of around 0.19 Å is obtained for **1** between its deposited and the re-refined structures. The coordinate positions of **2** are also improved overall, especially aminopiperidine motif and butynyl group positions (Fig 2B). The obtained rmsd value for **2,** between its deposited and the re-refined structures, is around 0.21 Å. In the complex structure involving **3**, positions of carbon (C9, C10) and nitrogen atoms (N13) of the aminopiperidine motif of **3** are improved (Fig 2C), reflecting in the calculated rmsd value (0.15 Å) for the inhibitor **3** between its deposited and the re-refined structures. The locations of carbon atoms (C15, C18) and nitrogen atom (N13) of the piperazine moiety in **4** are also moderately improved (Fig 2D). The calculated rmsd value between the deposited and the re-refined structures for this inhibitor **4** is 0.09 Å. Since some of these atoms or the neighboring ones may involve in the interaction with the amino acids, it is expected that these structural improvements through re-refining and re-building procedures would enhance the quality of the results of the subsequent FMO calculations. The rmsd values, between the deposited and the re-refined structures, for the twenty amino acids in the protein active site in all these four DPP-4 complex structures were also estimated and the obtained rmsd values were given in S1 Table of the supporting information.

### Notable IFIEs between inhibitors and DPP-4

Subsites in the active site of a protease are generally defined by the binding site of the substrate peptide [40]. The amino acid residues in the substrate peptide are numbered from the point of cleavage (P_2_, P_1_, $P_{1}^{'}$, $P_{2}^{'}$ …), and the protein subsites occupied by the respective amino acids are numbered in the same fashion (S_2_, S_1_, $S_{1}^{'}$, and $S_{2}^{'}$ …). In the present case, the N-terminus of the substrate peptide is recognized by Glu205, Glu206, and Ser630 cleaves at the N-terminus penultimate position (P_1_). We used the same definition for these four subsites in the present study. In addition to these four subsites, S_2_ extensive subsite, the site beyond S_2_ that is supposed to involve in the interaction, is defined as that by Nabeno et al. [10]. This site is composed of Val207, Ser209, Phe357, and Arg358.

The inhibitor binding leads to two important changes in the DPP-4 structure: (1) Tyr547 makes a conformational change, with the binding of **2** and **3**, so to have a *π*-*π* stacking interaction with the uracil moieties of **2** and **3**. No such conformational change in Tyr547 is noticed, however, with the binding to the other two inhibitors and (2) Arg358 goes for a side-chain reorientation with the binding of **1** and **4** that enables the opening of the S_2_ extensive subsite, in which trifluoromethyl and phenyl moieties of **1** and **4**, respectively, occupy. This paves the way for these two inhibitors to interact to S_2_ extensive subsite.

**Fig 4:**
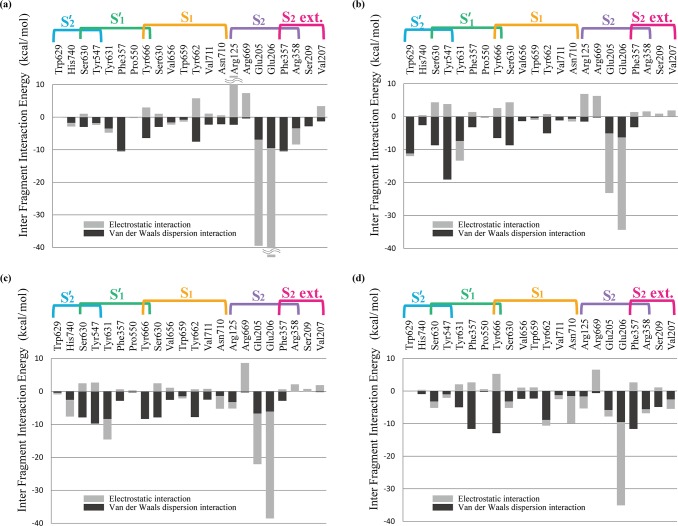
Interfragment interaction energy (IFIE) between amino acid residues at the active site of DPP-4 and the inhibitors [(a) sitagliptin **1**, (b) linagliptin **2**, (c) alogliptin **3**, (d) teneligliptin **4**]. The electrostatic (gray) and van der Waals (black) interaction energies are shown separately.

Fig 3 represents the binding mode of various inhibitors in the active site of the DPP-4. As shown in the Figs **2**and **3**interact to S_1_, S_2_, $S_{1}^{'}$, and $S_{2}^{'}$ subsites (Fig 3B and 3C), and **1** and **4** bind to S_1_, S_2_, $S_{1}^{'}$, and S_2_ extensive subsites (Fig 3A and 3D). Overall, these DPP-4 inhibitors reside inside the hydrophobic cavity made up of Arg125, Glu205, Glu206, Tyr547, Tyr662, Tyr666, Ser630, and Phe357.

Interfragment interaction energies calculated between the four selected inhibitors and several subsite residues of the DPP-4 are depicted in Fig 4A–4D. As it is mentioned in the Introduction section, both electrostatic and dispersion interactions can be studied and quantified separately using FMO calculations. It is known that DPP-4 inhibitors interact strongly with the Glu206 and Glu205 amino acids in the S_2_ subsite by forming salt bridges with them. The calculated IFIEs of these residues with all the inhibitors are large and especially Glu206 has the largest IFIE with all the considered inhibitors. One of the notable differences is that IFIE calculated between Glu205 and the inhibitor **4** is much smaller than those obtained between the same Glu and the other inhibitors. A close look at the complex structures reveals that inhibitors interact with these Glu205 and Glu206 amino acids by not only forming salt bridges but also effectively making hydrogen bonding with them and so this interaction should be considered as a combination of charge-charge and hydrogen bonding electrostatic interactions. In the case of Glu206 interacting with the inhibitors, both the oxygen atoms of its carboxyl group make strong hydrogen bonds (with 1.9 Å and 2.3 Å bond lengths) with the hydrogen atoms of the inhibitors; however, it is only one of the oxygen atoms, of the carboxyl group, of Glu205 forms a hydrogen bonding (1.8 Å) with the inhibitors. The other oxygen atom of this glutamic acid makes a strong hydrogen bonding (with 1.8 Å bond length) interaction with Arg125. This is one of the reasons for why inhibitors have larger IFIEs (mostly due to the electrostatic interaction) with Glu206 than those with Glu205. In the case of **4**, its interaction with Glu206 is similar to those of the other inhibitors, but its hydrogen bonding interaction with Glu205 is weaker (bond length of 2.5 Å), when compared with those of other inhibitors, and hence decrease in the calculated IFIE.

Let us see the nature of the other important interactions of DPP-4 with of its each inhibitors separately. In the case of **2**, its 2-butynyl group that resides in the S_1_ pocket interacts with Tyr662, Ser630, and Tyr666. As mentioned by Nabeno et al. [10], **2** interacts with Try547 through its uracil moiety by forming a *π*-*π* stacking (distance between the rings is 3.1 Å); however, in addition to this *π*-*π* interaction, its imidazole group also interacts with the same tyrosine residue through OH-*π* interaction (distance is 2.9 Å). This strong interaction reflects in the calculated IFIE value, -15.28 kcal/mol (van der Waals dispersion interaction amounts -19.08 kcal/mol and the repulsive electrostatic interaction is 3.80 kcal/mol, Fig 4B). Methylquinazolinone interacts strongly with Try629 (-11 kcal/mol) by not only making *π*-π interaction with both phenyl and pyrrole ring of the tryptophan through its phenyl component of the quinazoline moiety but also by interacting via CH-*π* interaction (2.7 Å) through its pyrimidine ring. In addition to the interaction with 2-butynyl group, Tyr666 also makes a CH-*π* interaction with the piperidine ring (2.7 Å). The overall IFIE calculated with this tyrosine is -3.90 kcal/mol (van der Waals dispersion interaction is -6.50 kcal/mol and the repulsive electrostatic interaction is 2.60 kcal/mol). The piperidine ring also interacts with Phe357 ring through CH-*π* interaction (3.0 Å with around -3 kcal/mol strength).

Interaction of **4** with the DPP-4 is interesting. Apart from the strong interaction with Glu206, it has one other significant hydrogen bonding. Its carbonyl group forms a strong hydrogen bonding with Asn710 (2.1 Å). Its thiazoline moiety interacts with Tyr662, and its pyrrolidine ring also interacts with the same tyrosine via hydrogen bonding. This combined interaction results in a larger IFIE (-10.60 kcal/mol, Fig 4D) than that seen in the case of **2** (Fig 4B). The other two main interactions come through Tyr666 and Phe357. The CH groups of thiazoline form CH-*π* interactions with Tyr666 ring and the overall calculated IFIE is -7.62 kcal/mol (van der Waals dispersion interaction -12.89 kcal/mol, electrostatic interaction 5.27 kcal/mol). The piperazine moiety of **4** interacts with Phe357 through CH-*π* mode and the IFIE is calculated to be -8.94 kcal/mol (with -11.61 kcal/mol contribution from van der Waals dispersion interaction and 2.68 kcal/mol through electrostatic interaction). The present study also showed that this inhibitor has moderate interactions with Arg358 (IFIE of -6.78 kcal/mol) and Ser209 (IFIE of -3.75 kcal/mol).

In the case of **3**, its cyanobenzyl moiety that resides in the subsite S_1_ interacts with Tyr662, Tyr666, and Ser630, and the calculated overall IFIEs are -7.10, -8.25, and -5.38 kcal/mol, respectively (Fig 4C). The uracil moiety makes a moderate *π*-*π* interaction with Tyr547 (3.4 Å), which accounts -9.70 kcal/mol energy and the overall IFIE with this tyrosine is calculated to be -7.00 kcal/mol. Inhibitor **3** interacts also with Tyr631 through its cyanobenzyl group (CH-*π*) as well through carbonyl group of its uracil moiety (hydrogen bonding).

Sitagliptin **1** forms the strongest bonding with Glu206 and Glu205 (Fig 4A) residues through its amine group (O····H bond lengths are as small as 1.7 and 1.8 Å). The present calculation reveals that its trifluorophenyl ring that binds to the S_1_ subsite has only moderate interactions with Tyr662 and Tyr666. The other strong interaction comes through Phe357 –the triazolopyrazine moiety *π*-*π* stacks with this phenylalanine and the calculated IFIE is -10.64 kcal/mol. CF_3_ group interacts moderately with Arg358.

### Comparison of IFIEs: linagliptin vs alogliptin and teneligliptin vs sitagliptin

On the basis of their binding subsites [10], **2** and **3** belong to Class II category (binds to S_1_, S_2_, $S_{1}^{'}$, and $S_{2}^{'}$ subsites) and **1** and **4** belong to Class III category (binds to S_1_, S_2_, $S_{1}^{'}$, and S_2_ extensive subsites).

Among the two xanthine-based inhibitor drugs, **2** showed 8-fold higher activity than **3**, and Nabeno et al. [10] noted that one of the reasons for this superior activity might be due to the fact that only **2** can engage interacting at the $S_{2}^{'}$ subsite. The present study supports this fact that phenyl component of the quinazolinone moiety (of **2**) makes a formidable interaction with Trp629 by forming π-π stacking with phenyl as well as with pyrrole ring of this tryptophan residue. In addition to this, the pyrimidine ring of this quinazolinone forms a CH-*π* interaction with this tryptophan and these combined interactions resulted into a strong binding of over -11 kcal/mol. This is completely absent in **3**. One other important factor that separates these two inhibitors is their interactions with Tyr547. While **3** interacts (*π*-*π* stacking) with Tyr547 through its uracil substructure, **2** interacts with the same tyrosine through both of its uracil (*π*-*π*) and imidazole (OH-*π*) moieties. This has been evidenced from the calculated hydrophobic IFIE values: -19.1 and -9.7 kcal/mol for **2** and **3**, respectively. All these factors might have caused the superior activity of **2**. It should be mentioned that the binding affinity of **2** towards DPP-4 is predicted to be higher than that of **3** [10, 41] and its IC_50_ value is smaller [12, 42]. Recent binding kinetics studies on these ligands using SPR have also supported this prediction [12]. Large binding affinity predicted for the inhibitor **2** might be attributed mainly to the unique existence of its strong interactions with Trp629 and Tyr547 residues at $S_{2}^{'}$ subsite.

The presence of the triazolopiperazine and piperazine moiety in **1** and **4**, respectively, at the P_2_ position makes **4** and **1** to engage in interaction at the S_2_ extensive subsite and introduces the “anchor lock domain” resulting in an increase of the binding activity owning to stronger hydrophobic interactions [10] at this subsite. As it has been mentioned earlier, **4** has the weakest interaction with Glu205, but it has 5-fold higher activity than **1**. So, this superior activity of **4** should have come from other quarters. It has been concluded in previous studies [10, 43] that interaction at S_2_ extensive subsite by **1** and **4** contributes to increase in their inhibitory activities. Among these two inhibitors, **4** has a larger contact area (0.92 nm^2^) in the S_2_ extensive subsite than **1** has (0.71 nm^2^) and, it has been concluded by Nabeno et al. [10] that the former may bind more tightly to this extensive subsite and this might be one of the reasons for its larger inhibitory activity. Though the present study supports this fact that the interaction of **4** with Phe357, Arg358, Ser209, and Val207 at this S_2_ extensive subsite is marginally stronger (Fig 4A & 4D), the difference in interaction energies is not a significant one to explain the higher inhibitory activity of **4**. Careful analysis reveals that it is not just the S_2_ extensive subsite but also S_1_ subsite that provides the room for **4** to have a stronger interaction with DPP-4: (1) its carbonyl group forms a strong hydrogen bond with Asn710 and (2) its interaction with Typ666 ring is doubly stronger (calculated hydrophobic interaction energy between **4** and **1** with Tyr666 is -12.89 and -6.40 kcal/mol, respectively).

Comparing **2** and **4** provides some interesting outcome: the main difference in their interaction pattern is that the former interacts strongly at $S_{2}^{'}$ subsite and the latter has some significant interactions at S_2_ extensive subsite. The IC_50_ value obtained for **2** is smaller than that obtained for **4** [12, 42]. This affords an assertion that strong hydrophobic interactions at $S_{2}^{'}$ subsite is more crucial, than the interactions at S_2_ extensive subsite, for an efficient DPP-4 inhibitor drug.

### Correlation between inhibitor activity and its hydrophobic IFIE with DPP-4

It should be mentioned that the calculated FMO-IFIE is not the difference between the energy of the protein–ligand complex and the sum of the energies of the ‘free’ protein and ligand but rather represents the “strength” of the interactions between the ligand and protein residues in the complex. The total IFIEs (sum of all the IFIEs) describe the stability of the protein-ligand complex. This stability correlates with, but is not the same as, the binding energy. By keeping this fact in mind, we compared the calculated IFIEs with the experimental binding energies and potencies to check whether there is any correlation between them.

**Fig 5:**
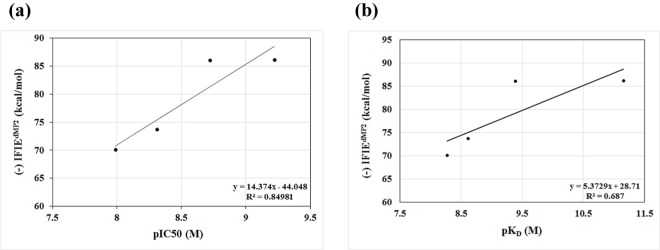
Relationship between hydrophobic IFIE (kcal/mol), calculated between the inhibitors and DPP-4, and (a) inhibitor activity [pIC50] (values taken from Ref. 10 and 42) and (b) experimental binding affinity [pK_D_] of the inhibitors from surface plasmon resonance (SPR) data (values taken Ref. 12).

Table 1 shows that number of non-hydrogen atoms of the inhibitors correlates well with the interfacial buried surface area (*R*^2^ = 0.95). These two factors correlate moderately with the calculated hydrophobic IFIEs (between DPP-4 and the inhibitors) with *R*^2^ value of 0.579 and 0.662, respectively. These observations are consistent with the trend predicted for the PPI inhibitors that the number of non-hydrogen atoms of these inhibitors correlates nicely with their binding energies with the proteins [44] in which the interactions are mainly composed by hydrophobic ones [45]. Notably, the calculated hydrophobic IFIEs of the inhibitors (with DPP-4) correlate well with their pIC_50_ values [10, 42] with *R*^2^ of 0.85 (Fig 5A). This outcome is coherent with the trend obtained for the PPI that their binding affinities correlate with their Van der Waals energies [46]. We also compared the calculated hydrophobic IFIEs with the recently [12] observed binding affinities, K_D_ from SPR data, of various inhibitor drugs to the DPP-4 (Fig 5B indicates the calculated IFIEs vs experimental pK_D_ values) and found a moderate correlation (*R*^2^ = 0.69). By considering the fact that the binding affinity is not only depending on specific interactions obtained by FMO but also depends on other factors such as entropy, desolvation, and strain energy present in the ligand’s bioactive conformation, this correlation could be considered as a significant one. These calculated hydrophobic IFIEs also have good correspondence with the SPR-derived off-rates and residence times [12]. All these observations reveal that hydrophobic interactions play an essential role in the inhibitory activities of these DPP-4 inhibitors. Subsequently, larger endogenous substrates, which could cover the sizeable DPP-4 active site of the hydrophobic pocket extensively, could provide increased inhibitory activities than by smaller inhibitors even with higher binding energies.

**Table 1:** Correlation between number of non-hydrogen atoms of ligands and the interfacial buried surface area with chain A of DPP-4. The correlation coefficient is 0.97.

	Number of non-hydrogen atoms	Interfacial buried surface area (Å^2^)
Sitagliptin **1**	28	265.9
Linagliptin **2**	35	318.3
Alogliptin **3**	25	233.6
Teneligliptin **4**	30	293.9

## Conclusions

Through the present investigation, interaction between the DPP-4 and its inhibitor drugs were quantitatively studied by using quantum mechanical fragment molecular orbitals method at correlated RI-MP2/cc-pVDZ level. The present residue-level interaction study reveals that though all the inhibitors generally form strong interactions with Glu205 and Glu206 amino acids through forming salt bridges as well as by effectively making hydrogen bonds with them, the effectual interactions exist at $S_{2}^{'}$ subsite, for the linagliptin **2** and alogliptin **3**, and S_2_ extensive and S_1_ subsites, for sitagliptin **1** and teneligliptin **4**. The superior inhibitory activities of **2** and **4** were thoroughly investigated by elaborately studying the interaction between the DPP-4 and its inhibitors. Hydrophobic IFIEs correlate well with the activities of the DPP-4 inhibitors. The study concludes that interactions, especially the hydrophobic ones, at the $S_{2}^{'}$, S_2_ extensive, and S_1_ subsites are crucial. Among others, interactions with Try547 and Trp629, for the category II inhibitors, and with Phe357 and Tyr666, for the category III inhibitors, are decisive for their DPP-4 inhibitory activities.

Hence, inhibitors covering the DPP-4 active site significantly interact extensively with the hydrophobic pocket by their hydrophobic inhibitor moieties. The cumulative strength of these hydrophobic interactions becomes large for these inhibitors and therefore linagliptin and teneligliptin have larger interaction energies, and consequently higher inhibitory activities, than their alogliptin and sitagliptin counterparts. The present conclusions can be used effectively to search novel new inhibitors of DPP-4.

## Supporting Information

S1 Table Root mean square deviations (rmsd) between deposited and re-refined structures of 20 amino acid residues in the active site of DPP-4 complexes. All values are in Å.
(DOCX)
