# Supplementary material for: Comparative Binding Analysis of Dipeptidyl Peptidase IV (DPP-4) with Antidiabetic Drugs – An Ab Initio Fragment Molecular Orbital Study
Source: PLoS One. 2016 Nov 10;11(11):e0166275. doi: 10.1371/journal.pone.0166275 (PMC5104442; doi:10.1371/journal.pone.0166275)
Supplement: S1 Table — All values are in Å. (DOCX) [file pone.0166275.s001.docx]

**S1 Table. Root mean square deviations (rmsd) between deposited and re-refined structures of 20 amino acid residues in the active site of DPP-4 complexes. All values are in Å.**

|  |  |  |  |  |
| --- | --- | --- | --- | --- |
| Residues | Sitagliptin **1** | Linagliptin **2** | Alogliptin **3** | Teneligliptin **4** |
|  |  |  |  |  |
| Trp629 | 0.05 | 0.07 | 0.04 | 0.02 |
| His740 | 0.02 | 0.06 | 0.06 | 0.05 |
| Ser630 | 0.10 | 0.03 | 0.04 | 0.02 |
| Tyr547 | 0.08 | 0.10 | 0.07 | 0.03 |
| Tyr631 | 0.03 | 0.14 | 0.05 | 0.04 |
| Phe357 | 0.06 | 0.12 | 0.03 | 0.03 |
| Pro550 | 0.20 | 0.09 | 0.22 | 0.02 |
| Tyr666 | 0.05 | 0.07 | 0.03 | 0.03 |
| Val656 | 0.08 | 0.04 | 0.02 | 0.04 |
| Trp659 | 0.07 | 0.06 | 0.04 | 0.03 |
| Tyr662 | 0.05 | 0.05 | 0.03 | 0.05 |
| Val711 | 0.06 | 0.04 | 0.04 | 0.03 |
| Asn710 | 0.06 | 1.11 | 0.06 | 0.04 |
| Arg125 | 0.29 | 0.14 | 0.10 | 0.13 |
| Arg669 | 0.05 | 1.01 | 0.05 | 0.05 |
| Glu205 | 0.07 | 0.10 | 1.03 | 0.03 |
| Glu206 | 0.06 | 0.09 | 0.03 | 0.08 |
| Arg358 | 0.13 | 0.81 | 0.08 | 0.08 |
| Ser209 | 0.05 | 0.09 | 0.04 | 0.04 |
| Val207 | 0.05 | 0.07 | 0.03 | 0.03 |
|  |  |  |  |  |
